# Are the Hypertrophic Adaptations to High and Low-Load Resistance Training Muscle Fiber Type Specific?

**DOI:** 10.3389/fphys.2018.00402

**Published:** 2018-04-18

**Authors:** Jozo Grgic, Brad J. Schoenfeld

**Affiliations:** ^1^Institute of Sport, Exercise and Active Living, Victoria University, Melbourne, VIC, Australia; ^2^Department of Health Sciences, Lehman College, Bronx, NY, United States

**Keywords:** musculoskeletal, resistance, exercise, loading, muscles, growth

## Introduction

Skeletal muscle displays considerable biochemical complexity, physiological plasticity, and cellular heterogeneity (Ohlendieck, 2010). It is well-recognized that resistance training (RT) is the most potent non-pharmacological interventional strategy for achieving increases in skeletal muscle size (American College of Sports Medicine, 2009). The American College of Sports Medicine (2009) recommends RT in the 6–12 repetition maximum (RM) range as being ideal for increases in muscle hypertrophy. However, Schoenfeld et al. (2017) concluded that, based on the current evidence, low-load (≤60% 1 RM) RT performed to momentary muscular failure increases muscle size in a manner similar to high-load (>60% 1 RM) RT. It should be noted that these conclusions were specific to whole muscle imaging techniques (i.e., ultrasound, magnetic resonance imaging, and computerized tomography); a meta-analysis for direct histological determination (i.e., muscle biopsy) could not be performed due to the lack of studies meeting the inclusion criteria of the review.

The evidence is equivocal regarding the agreement between whole muscle imaging techniques and histological determination of muscle hypertrophy. For instance, McCall et al. (1996) reported a 13% increase in muscle cross-sectional area (CSA) as measured via magnetic resonance imaging, along with a 10 and 17% increase for type I and type II muscle fibers, respectively. While the meta-analysis of Schoenfeld et al. (2017) reported similar changes in muscle size irrespective of loading schemes, the question remains as to whether the hypertrophy of type I and type II muscle fibers was also similar between high- and low-load conditions. It might be that high-load RT emphasizes type II muscle hypertrophy (Campos et al., 2002), with low-load RT stimulating greater growth in type I muscle fibers (Ogborn and Schoenfeld, 2014; Grgic et al., 2018).

A number of acute electromyography (EMG) studies show that EMG amplitude is significantly greater with high- vs. low-load RT thereby suggesting that higher loads are needed to fully stimulate the highest threshold motor units associated with type IIx fibers (Schoenfeld et al., 2014, 2016). However, greater EMG amplitude in a given condition does not necessarily reflect long-term adaptations to regimented RT (for a detailed review on the topic see Vigotsky et al., 2018). The only way to assess this topic is by analyzing studies that directly compared high-load and low-load RT and their impact on muscle fiber hypertrophy. Therefore, the present article aims to discuss and interpret the studies that assessed the muscle fiber changes that occur longitudinally with high- and low-load RT schemes. While acknowledging that there are several hybrid type fibers delineated in human skeletal muscle, the basic classification to type I, type IIa, and type IIx will be used for this paper.

## The effects of high vs. low-load resistance training on muscle fiber hypertrophy adaptations

Only a few studies have investigated this topic thus far (Table 1). Jackson et al. (1990) conducted a counterbalanced crossover trial in which participants first performed a 7.5-week mesocycle of high-load muscular strength-oriented RT or 7.5 weeks of low-load muscular endurance-oriented RT. Following a 5.5-week “washout” period (i.e., no RT performed), participants crossed over to perform the alternative routine. After the first 7.5 weeks, an increase in the size of all muscle fiber types for both RT groups occurred. After the second 7.5 weeks of RT, the participants performing the muscular strength mesocycle as their second treatment reported a further growth of type I and II fibers. By contrast, those that performed the muscular endurance-oriented RT for their second treatment showed a decrease in the size of all fiber types, with the greatest decrease noted for type IIx fibers. These findings suggest that a progression from low to high-loads might result in gains that are more consistent.

**Table 1:** Summary of the findings from the studies comparing high and low-load training and its effects on muscle fiber hypertrophy adaptations.

**Reference**	**Study design**	**Resistance training exercise(s)**	**Training frequency and study duration**	**Method of fiber typing**	**Results**	Relative effects (%)^*^
Campos et al., 2002	Young untrained men (*n* = 27) were allocated either to a high-load group (3–5 RM) a moderate-load (9–11 RM) or a low-load group (20–28 RM).	Squat, leg press, and knee extension	2–3 times per week; 8 weeks	mATPase histochemistry	Significant pre- to post-intervention increases in type I, type IIa and type IIx muscle fibers were reported in the high-load and moderate-load groups. No significant increases across muscle fiber types were reported for the low-load group.	Type I fibers: High-load = +12 Moderate-load = +13 Low-load = +10 Type IIa fibers: High-load = +23 Moderate-load = +16 Low-load = +8 Type IIx fibers: High-load = +25 Moderate-load = +27 Low-load = +14
Dons et al., 1979	Young untrained men (*n* = 12) were allocated either to a high-load group (80% 1 RM) or a low-load group (50% 1 RM).	Squat	3 times per week; 7 weeks	mATPase histochemistry	No significant pre- to post-intervention increases in muscle fiber size were reported.	Not presented
Jackson et al., 1990	Young untrained men (*n* = 12) were allocated either to a high-load group (4 RM) or a low-load group (15–25% 1 RM).	Knee extension	4 times per week; 7.5 weeks	mATPase histochemistry	Both high-load and low-load groups increased the size of all muscle fiber types following the first treatment. Participants that performed high-load training as their second treatment showed further increases in the size of type I and type IIx muscle fibers. In contrast, those performing low-load training as their second treatment showed decreases in the size of all fiber types.	Not presented
Lamas et al., 2010	Young untrained men (*n* = 22) were allocated either to a high-load group (4–10 RM) or a low-load group (30–60% 1 RM).	Squat	3 times per week; 8 weeks	mATPase histochemistry	Significant pre- to post-intervention increases in type IIa, and type IIx muscle fibers both for the high-load and low-load groups with no significant between-group differences. The high-load group increased type I muscle fiber size, in contrast to the low-load group, in which, a decrease in the size of type I muscle fiber was reported.	Type I fibers: High-load = +15 Low-load = −5 Type IIa fibers: High-load = +18 Low-load = +15 Type IIx fibers: High-load = +41 Low-load = +19
Mackey et al., 2011	Young untrained men (*n* = 12) were allocated either to a high-load group (70% 1 RM) or a low-load group (15.5% 1 RM).	Knee extension	3 times per week; 12 weeks	mATPase histochemistry	No significant pre- to post-intervention increases in muscle fiber size were reported in either group.	Type I fibers: High-load = −2 Low-load = +1 Type II fibers: High-load = −1 Low-load = −5
Mitchell et al., 2012	Young untrained men (*n* = 18) were allocated either to one of two high-load groups (80% 1 RM) or a low-load group (30% 1 RM).	Knee extension	3 times per week; 10 weeks	mATPase histochemistry	Significant pre- to post-intervention increases in the size of both type I and type II muscle fibers, with no significant between-group differences.	Type I fibers: High-load = +16 High-load = +14 Low-load = +23 Type II fibers: High-load = +16 High-load = +18 Low-load = +12
Morton et al., 2016	Young trained men (*n* = 49) were allocated to a high-load group (75–90% 1 RM) or a low-load group (25–50% 1 RM).	Seated row, bench press, shoulder press, front plank, bicep curls, triceps extension, wide grip pull-downs, leg press, knee extension, and knee flexion.	4 times per week; 12 weeks	Antibody method	Significant pre- to post-intervention increases in the size of both type I and type II muscle fibers, with no significant between-group differences	Type I fibers: High-load = +13 Low-load = +11 Type II fibers: High-load = +18 Low-load = +14
Netreba et al., 2013	Young untrained men (*n* = 14) were allocated either to a high-load group (85–90% 1 RM) a moderate-load (60–70% 1 RM) or a low-load group (20–25% 1 RM).	Leg press	3 times per week; 8 weeks	Antibody method	In the high-load groups, significant pre- to post-intervention increases in all muscle fiber types occurred. In the low-load group, significant pre- to post-intervention increases occurred in type I but not in type II muscle fibers. The high-load group increased the size of type II muscle fibers to a greater extent than the low-load group. The moderate-load group increased the size of both fiber types equally. The low-load group increased type I muscle fibers to a greater extent than the high-load group.	Type I fibers: High-load = +10 Moderate-load = +11 Low-load = +18 Type II fibers: High-load = +20 Moderate -load = +13 Low-load = +8
Schuenke et al., 2012	Young untrained women (*n* = 27) were allocated either to a high-load group (6–10 RM), high-load, low-velocity group (6–10 RM) or a low-load group (20–30 RM).	Squat, leg press, and knee extension	2–3 days per week; 6 weeks	mATPase histochemistry	Significant pre- to post-intervention increases in type I, type IIA and type IIX muscle fibers for the high-load group. Significant pre- to post-intervention increases in type IIa, and type IIx muscle fibers were reported for the high-load, low-velocity group. No significant increases in muscle fibers were reported for the low-load group.	Type I fibers: High-load = +26 High-load low-velocity = +6 Low-load = 0 Type IIa fibers: High-load = +31 High-load low-velocity = +12 Low-load = +9 Type IIx fibers: High-load = +37 High-load low-velocity = +19 Low-load = +9
Taaffe et al., 1996	Older untrained women (*n* = 14) were allocated either to a high-load group (80% 1 RM) or a low-load group (40% 1 RM).	Leg press, knee flexion, and knee extension	3 days per week; 52 weeks	mATPase histochemistry	Significant pre- to post-intervention increases in type I, muscle fibers for the high-load and low-load group. No significant pre- to post-intervention increases were reported for type II muscle fibers.	Type I fibers: High-load = +28 Low-load = +10 Type II fibers: High-load = +22 Low-load = +18
Vinogradova et al., 2013	Young untrained men (*n* = 60) were allocated either to one of three high-load groups (80–85 % 1 RM) one moderate-load group (65–70% 1 RM) or one of two low-load groups (50% 1 RM).	Leg press	Training frequency was not presented; 8–10 weeks	Antibody method	Significant pre- to post-intervention increases in type II muscle fibers for the high-load groups. Significant pre to post-intervention increases in type I muscle fibers for the moderate-load and low-load groups.	Type I fibers: High-load = +9 High-load = +6 High-load = +6 Moderate-load = +18 Low-load = +18 Low-load = +18 Type II fibers: High-load = +23 High-load = +20 High-load = +33 Moderate-load = +6 Low-load = +8 Low-load = +6

* *Some studies presented only values for II muscle fibers without further typing to type IIa and IIx; RM, repetition maximum*.

Only one study was performed in older adults. Taaffe et al. (1996) sought to compare the effects of high- vs. low-load RT on muscle size using an intervention spanning 1 year. Untrained women exercised 3 days per week, whereby the high-load group performed ten repetitions with external loads of 80% 1 RM while the low-load group performed 14 repetitions with loads corresponding to 40% 1 RM. When evaluating percent changes, the high-load group achieved greater hypertrophy in both type I and type II muscle fibers. On the surface, these findings suggest that high-load RT is a prerequisite for maximizing hypertrophy across fiber types. However, the researchers opted to equate the total volume load between conditions by having the low-load group stop each set well short of volitional fatigue. Such events inadvertently bias results in favor of the high-load condition given that a high level of fatigue is obligatory to achieve hypertrophic benefits when training with lower loads (Morton et al., 2016).

Of crucial importance is that in both of the studies mentioned above, the RT program in the low-load groups was not performed to momentary muscular failure, indicating unequal training stimuli between high and low-load conditions. Employing a low-load RT program and not performing repetitions to momentary muscular failure may hinder muscular adaptation (Burd et al., 2012). According to Henneman (1985) size principle, larger motor units will be sequentially recruited as force production requirements increase, ultimately resulting in activation of the entire motor unit pool. It is possible that with low-load training, the lower threshold motor units will be under load for a longer period, which in turn, might augment the hypertrophic response of type I muscle fibers. This effect might not occur with high-load training, and possibly because of this, preferential hypertrophy of type II muscle fibers has been commonly reported with RT (Folland and Williams, 2007).

Campos et al. (2002) were the first to compare high- vs. low-load RT with both groups training to momentary muscular failure while using histological measures of muscle growth. The researchers' randomized 27 untrained participants into three different loading groups: high-load (3–5 RM), moderate-load (9–11 RM) and low-load (20–28 RM) RT programs. After 8 weeks of RT, all muscle fibers types hypertrophied in the high- and moderate-load groups. However, no significant pre- to post-intervention increase in muscle fCSA was noted in type I or type II muscle fibers for the low-load group. Schuenke et al. (2012) reported similar results to that of Campos et al. (2002). After 6 weeks of lower body RT, the low-load group (40–60% 1 RM) showed no significant increases in type I muscle fiber CSA while the high-load group (80–85% 1 RM) achieved robust increases in the size of all muscle fiber types, with the greatest gains observed in type IIx fibers. Although muscle tissue is a prime example of tissue plasticity and capable of undergoing dramatic changes in phenotypic profile with regimented RT, the rate of increases in muscle fiber size for the high-load condition deserves scrutiny (Burd et al., 2013). The researchers reported a muscle fiber growth rate of 0.66% per day of training, which is ~ 4- to 5-fold larger than the values presented in the review by Wernbom et al. (2007). Still, while not as large as found by Schuenke et al. (2012) similar growth rates with high-load lower body RT have been noted previously in the literature (Staron et al., 1990; Lamas et al., 2010).

Interestingly, some studies report that low-load RT induces a greater hypertrophic response in type I muscle fibers. Vinogradova et al. (2013) compared high- (80–85% of 1 RM) vs. low-load (50% 1 RM) RT in a group of untrained young men. The results indicated that the growth of muscle fiber types is directly related to the training load. Specifically, the high-load group achieved the greatest increases in type II muscle fiber size whereas the low-load group achieved the highest increases in type I muscle fiber size. The researchers hypothesized that the greater increases in type I muscle fibers for the low-load group were related to greater metabolic stress (Vinogradova et al., 2013). Metabolic stress relates to the build-up of metabolites, for example increases in calcium flux, lactate, potassium and hydrogen ions, and is a mechanism hypothesized to mediate muscle hypertrophy via increased fiber recruitment, changes in hormonal production, and/or cell swelling, among others (Schoenfeld, 2013). Similar findings to those presented by Vinogradova et al. (2013) have been reported by the same laboratory, with low-load RT leading to greater increases in type I hypertrophy and high-load RT enhancing type II fiber hypertrophy (Netreba et al., 2013).

Using a within-subject design, Mitchell et al. (2012) randomly assigned 18 men in counterbalanced fashion to performing unilateral knee extension with one leg training at high-loads (80% 1 RM) and the other at low-loads (30% 1 RM). After 10 weeks of RT, significant hypertrophy was noted from baseline to post-intervention in all muscle fiber types for both high-load (80% of 1 RM) and low-load (30% of 1 RM) RT groups. No statistically significant differences between loading conditions in muscle fiber growth were noted. Nonetheless, it is important to point out that the low-load group achieved a 23% increase in muscle type I muscle fiber size, in comparison to a 16% increase in type I muscle fiber size observed in the high-load group. Given the small sample size employed, this raises the possibility of a type 2 error whereby significant differences between conditions did in fact exist and, considering the magnitude of differences, that findings may be practically meaningful.

A common limitation in all of the studies above is the use of untrained participants. It has been shown that individuals with higher RT experience a blunted protein synthetic response post-resistance exercise, suggesting a possible ceiling effect of muscle gain (Damas et al., 2015). To address this gap in the literature, Morton et al. (2016) randomized young men with an average of 4 years RT experience to a 12-week total body exercise program using either a high-load (8–12 RM) or low-load (20–25 RM) scheme. Following the RT protocol, similar rates of growth were noted in all muscle fiber types regardless of the load condition. These isolated findings suggest trained individuals may experience equal growth across all fiber types regardless of the loading scheme used.

As with most RT research, the vast majority of studies that investigated this topic were of relatively short duration; it is not clear whether potential differences in fiber type hypertrophy specific to a given loading scheme may widen or narrow over time. Finally, all of the included studies assessed fiber growth in the vastus lateralis muscle. As previously highlighted, the vastus lateralis muscle is the most common muscle of choice for biopsies because of its mixed fiber type composition, accessibility, and trainability (Staron et al., 2000). Given the scarcity of data, the extrapolation of findings to other muscle groups is limited.

### Experimental considerations

The examination of fiber type changes via muscle biopsy has some inherent limitations. First, the biopsy technique involves extracting a small amount of tissue from a given muscle, which may not necessarily reflect fiber type-specific changes at the whole muscle level. Moreover, variations exist in the distribution of fiber types from superficial to deep and proximal to distal (Blomstrand and Ekblom, 1982), thus making it important to consider these factors when extrapolating findings into practical terms. Second, the method used to determine fiber types varies between studies. A majority of the reviewed literature employed mATPase histochemistry to delineate fiber typing, whereas several others (Netreba et al., 2013; Vinogradova et al., 2013; Morton et al., 2016) used antibodies, which may have greater accuracy in delineating hybrid fibers. Finally, differences in measurement of fCSA can result in differences in size estimates, thus limiting a between-study comparison.

## Conclusions

When low-load RT is not carried out to muscular failure, high-load training appears to provide a superior hypertrophic stimulus and thus greater growth of all muscle fibers. While some evidence indicates that low-load RT, when carried out to muscle failure, may induce a greater hypertrophic response in type I muscle fibers compared to high-load RT and that high-load RT may induce preferential growth of type II muscle fibers, the body of literature remains somewhat equivocal on the topic. In summary, there currently is not enough evidence to make a firm conclusion regarding changes that occur at the muscle fiber level with different loading schemes.

## Author contributions

All authors listed have made a substantial, direct and intellectual contribution to the work, and approved it for publication.

### Conflict of interest statement

The authors declare that the research was conducted in the absence of any commercial or financial relationships that could be construed as a potential conflict of interest.

## References

[B1] American College of Sports Medicine (2009). American College of Sports Medicine position stand. Progression models in resistance training for healthy adults. Med. Sci. Sports Exerc. 41, 687–708. 10.1249/MSS.0b013e3181915670 19204579

[B2] Blomstrand E. Ekblom B. (1982). The needle biopsy technique for fibre type determination in human skeletal muscle–a methodological study. Acta Physiol. Scand. 116, 437–442. 10.1111/j.1748-1716.1982.tb07163.x 6221506

[B3] Burd N. A. Andrews R. J. West D. W. Little J. P. Cochran A. J. Hector A. J.. (2012). Muscle time under tension during resistance exercise stimulates differential muscle protein sub-fractional synthetic responses in men. J. Physiol. 590, 351–362. 10.1113/jphysiol.2011.221200 22106173 PMC3285070

[B4] Burd N. A. Moore D. R. Mitchell C. J. Phillips S. M. (2013). Big claims for big weights but with little evidence. Eur. J. Appl. Physiol. 113, 267–268. 10.1007/s00421-012-2527-1 23086296

[B5] Campos G. E. Luecke T. J. Wendeln H. K. Toma K. Hagerman F. C. Murray T. F.. (2002). Muscular adaptations in response to three different resistance training regimens: specificity of repetition maximum training zones. Eur. J. Appl. Physiol. 88, 50–60. 10.1007/s00421-002-0681-6 12436270

[B6] Damas F. Phillips S. Vechin F. C. Ugrinowitsch C. (2015). A review of resistance training-induced changes in skeletal muscle protein synthesis and their contribution to hypertrophy. Sports Med. 45, 801–807. 10.1007/s40279-015-0320-0 25739559

[B7] Dons B. Bollerup K. Bonde-Petersen F. Hancke S. (1979). The effect of weight-lifting exercise related to muscle fiber composition and muscle cross-sectional area in humans. Eur. J. Appl. Physiol. Occup. Physiol. 40, 95–106. 10.1007/BF00421155 428373

[B8] Folland J. P. Williams A. G. (2007). The adaptations to strength training: morphological and neurological contributions to increased strength. Sports Med. 37, 145–168. 10.2165/00007256-200737020-00004 17241104

[B9] Grgic J. Homolak J. Mikulic P. Botella J. Schoenfeld B. J. (2018). Inducing hypertrophic effects of type I skeletal muscle fibers: a hypothetical role of time under load in resistance training aimed at muscular hypertrophy. Med. Hypotheses. 112, 40–42. 10.1016/j.mehy.2018.01.012 29447936

[B10] Henneman E. (1985). The size-principle: a deterministic output emerges from a set of probabilistic connections. J. Exp. Biol. 115, 105–112. 3161974 10.1242/jeb.115.1.105

[B11] Jackson C. G. Dickinson A. L. Ringel S. P. (1990). Skeletal muscle fiber area alterations in two opposing modes of resistance-exercise training in the same individual. Eur. J. Appl. Physiol. Occup. Physiol. 61, 37–41. 10.1007/BF00236691 2149702

[B12] Lamas L. Aoki M. S. Ugrinowitsch C. Campos G. E. Regazzini M. Moriscot A. S.. (2010). Expression of genes related to muscle plasticity after strength and power training regimens. Scand. J. Med. Sci. Sports 20, 216–225. 10.1111/j.1600-0838.2009.00905.x 19422645

[B13] Mackey A. L. Holm L. Reitelseder S. Pedersen T. G. Doessing S. Kadi F.. (2011). Myogenic response of human skeletal muscle to 12 weeks of resistance training at light loading intensity. Scand. J. Med. Sci. Sports 21, 773–782. 10.1111/j.1600-0838.2010.01178.x 21143306

[B14] McCall G. E. Byrnes W. C. Dickinson A. Pattany P. M. Fleck S. J. (1996). Muscle fiber hypertrophy, hyperplasia, and capillary density in college men after resistance training. J. Appl. Physiol. (1985) 81, 2004–2012. 10.1152/jappl.1996.81.5.2004 8941522

[B15] Mitchell C. J. Churchward-Venne T. A. West D. D. Burd N. A. Breen L. Baker S. K. (2012). Resistance exercise load does not determine training-mediated hypertrophic gains in young men. J. Appl. Physiol. (1985) 113, 71–77. 10.1152/japplphysiol.00307.2012 22518835 PMC3404827

[B16] Morton R. W. Oikawa S. Y. Wavell C. G. Mazara N. McGlory C. Quadrilatero J.. (2016). Neither load nor systemic hormones determine resistance training-mediated hypertrophy or strength gains in resistance-trained young men. J. Appl. Physiol. (1985) 121, 129–138. 10.1152/japplphysiol.00154.2016 27174923 PMC4967245

[B17] Netreba A. Popov D. Bravyy Y. Lyubaeva E. Terada M. Ohira T.. (2013). Responses of knee extensor muscles to leg press training of various types in human. Ross. Fiziol. Zh. Im. I. M. Sechenova. 99, 406–416. 23789443

[B18] Ogborn D. Schoenfeld B. J. (2014). The role of fiber types in muscle hypertrophy: implications for loading strategies. Strength Cond. J. 36, 20–25. 10.1519/SSC.0000000000000030

[B19] Ohlendieck K. (2010). Proteomics of skeletal muscle differentiation, neuromuscular disorders and fiber aging. Expert Rev. Proteomics 7, 283–296. 10.1586/epr.10.2 20377394

[B20] Schoenfeld B. J. (2013). Potential mechanisms for a role of metabolic stress in hypertrophic adaptations to resistance training. Sports Med. 43, 179–194. 10.1007/s40279-013-0017-1 23338987

[B21] Schoenfeld B. J. Contreras B. Vigotsky A. D. Ogborn D. Fontana F. Tiryaki-Sonmez G. (2016). Upper body muscle activation during low-versus high-load resistance exercise in the bench press. Isokinet. Exerc. Sci. 24, 217–224. 10.3233/IES-160620

[B22] Schoenfeld B. J. Contreras B. Willardson J. M. Fontana F. Tiryaki-Sonmez G. (2014). Muscle activation during low-versus high-load resistance training in well-trained men. Eur. J. Appl. Physiol. 114, 2491–2497. 10.1007/s00421-014-2976-9 25113097

[B23] Schoenfeld B. J. Grgic J. Ogborn D. Krieger J. W. (2017). Strength and hypertrophy adaptations between low- versus high-load resistance training: a systematic review and meta-analysis. J. Strength Cond. Res. 31, 3508–3523. 10.1519/JSC.0000000000002200 28834797

[B24] Schuenke M. D. Herman J. R. Gliders R. M. Hagerman F. C. Hikida R. S. Rana S. R.. (2012). Early-phase muscular adaptations in response to slow-speed versus traditional resistance-training regimens. Eur. J. Appl. Physiol. 112, 3585–3595. 10.1007/s00421-012-2339-3 22328004

[B25] Staron R. S. Hagerman F. C. Hikida R. S. Murray T. F. Hostler D. P. Crill M. T.. (2000). Fiber type composition of the vastus lateralis muscle of young men and women. J. Histochem. Cytochem. 48, 623–629. 10.1177/002215540004800506 10769046

[B26] Staron R. S. Malicky E. S. Leonardi M. J. Falkel J. E. Hagerman F. C. Dudley G. A. (1990). Muscle hypertrophy and fast fiber type conversions in heavy resistance-trained women. Eur. J. Appl. Physiol. Occup. Physiol. 60, 71–79. 10.1007/BF00572189 2311599

[B27] Taaffe D. R. Pruitt L. Pyka G. Guido D. Marcus R. (1996). Comparative effects of high- and low-intensity resistance training on thigh muscle strength, fiber area, and tissue composition in elderly women. Clin. Physiol. 16, 381–392. 10.1111/j.1475-097X.1996.tb00727.x 8842574

[B28] Vigotsky A. D. Halperin I. Lehman G. J. Trajano G. S. Vieira T. M. (2018). Interpreting signal amplitudes in surface electromyography studies in sport and rehabilitation sciences. Front. Physiol. 8:985. 10.3389/fphys.2017.00985 29354060 PMC5758546

[B29] Vinogradova O. L. Popov D. V. Netreba A. I. Tsvirkun D. V. Kurochkina N. S. Bachinin A. V.. (2013). Optimization of training: development of a new partial load mode of strength training. Hum. Physiol. 39, 71–85. 10.1134/S0362119713050162 25509874

[B30] Wernbom M. Augustsson J. Thomeé R. (2007). The influence of frequency, intensity, volume and mode of strength training on whole muscle cross-sectional area in humans. Sports Med. 37, 225–264. 10.2165/00007256-200737030-00004 17326698

